# Ergot Alkaloids in Feed for Pekin Ducks: Toxic Effects, Metabolism and Carry Over into Edible Tissues

**DOI:** 10.3390/toxins7062006

**Published:** 2015-06-02

**Authors:** Sven Dänicke

**Affiliations:** Institute of Animal Nutrition, Friedrich-Loeffler-Institute (FLI), Federal Research Institute for Animal Health, Bundesallee 50, 38116 Braunschweig, Germany; E-Mail: sven.daenicke@fli.bund.de; Tel.: +49-531-5804-4101; Fax: +49-531-5804-4299

**Keywords:** ergot alkaloids, carry over, Pekin duck, feed intake, clinical chemistry

## Abstract

Hardened sclerotia (ergots) of *Claviceps purpurea* contaminate cereal grains and contain toxic ergot alkaloids (EA). Information on EA toxicity in ducks is scarce. Therefore, the aim of the growth experiment (Day 0–49, *n* = 54/group) was to titrate the lowest observed adverse effect level (LOAEL) for total ergot alkaloids (TEA). A control diet was prepared without ergots, and the diets designated Ergot 1 to 4 contained 1, 10, 15 and 20 g ergot per kg diet, respectively, corresponding to TEA contents of 0.0, 0.6, 7.0, 11.4 and 16.4 mg/kg. Sensitivity of ducks to EA was most pronounced at the beginning of the experiment when feed intake decreased significantly by 9%, 28%, 41% and 47% in groups Ergot 1 to 4, respectively, compared to the control group. The experiment was terminated after two weeks for ducks exposed to Ergot 3 and 4 due to significant growth retardation. Ergot alkaloid residues in edible tissues were lower than 5 ng/g. Bile was tested positive for ergonovine (=ergometrine = ergobasine) with a mean concentration of 40 ng/g. Overall, the LOAEL amounted to 0.6 mg TA/kg diet suggesting that ducks are not protected by current European Union legislation (1 g ergot/kg unground cereal grains).

## 1. Introduction

Ergot alkaloids are the etiologic compounds responsible for the classical signs of ergotism in humans and animals [1,2,3] associated with the presence of ergot as the hardened mycelium of *Claviceps purpurea* in food and feed. Toxicologically, ergot alkaloids potentially interact with serotoninergic, dopaminergic and adrenergic receptors depending on their specific chemical structures [3]. Moreover, the combined overall toxic effects of differentially acting individual alkaloids further depend on their absolute concentrations and proportions to each other [3]. In addition, the total ergot alkaloid (TEA) content of sclerotia from *C. purpurea* varies largely between nearly zero to approximately 10,400 mg/kg (=1.04%) depending on geographic region and harvesting year, cereal species, variety and genotype [2,4,5]. This tremendous variation raises questions regarding the reliability of the current European Union (EU) regulation regarding the upper limit of 1000 mg ergot (*C. purpurea*) per kg unground cereal grains (=0.1%) as specified by Directive 2002/32/EC for animal health protection. Dietary levels of 0.3% ergot caused increased mortality in broilers while in another experiment ergot levels of 0.7% were without adverse effects. This apparent contradiction might be due to different TEA and support the view that variation in TEA content of ergot clearly determines its toxic effects [3]. Consequently, EFSA recommended replacing the physical method by chemical analysis. Furthermore, dose response experiments are required where a detailed ergot alkaloid analysis of feed is correlated to toxic effects and to a possible alkaloid transfer into edible tissues (carry-over), especially in farm animal species hitherto not or only rarely considered, such as ducks. Based on their potential interference with neurotransmitters and on the observation that voluntary feed intake is often affected by ergot in livestock without showing typical signs of classical ergotism makes feed intake and its regulation a suitable endpoint to study ergot effects.

Therefore, the aim of our study is to test increasing TEA concentrations in diets for growing Pekin ducks on voluntary feed intake, growth performance, general health and carry-over of ergot alkaloids into edible tissues and other samples such as blood or bile.

## 2. Results

### 2.1. Composition of Ergoty Rye and Experimental Diets

The ergoty rye batch was composed of 45.2% ergot and 54.8% rye and contained approximately 18% crude protein, 20% crude fiber and 32% crude fat (Table 1). The proportion of the ergot-specific fatty acid ricinoleic acid (12–OH–C18:1) of the crude fat fraction amounted to 7.4% and corresponded to 24 g/kg of the ergoty rye. Considering the proportions of the ergoty rye of the whole diets the final calculated concentrations of ricinoleic acid in control and Ergot 1 to 4 diets were 0.0, 0.1, 0.5, 0.8 and 1.1 g/kg, respectively. The TEA content of the ergoty rye amounted to 436 mg/kg (Table 1). The proportion of key alkaloids (=sum of those alkaloids for which analytical standards were commercially available, *i.e.*, ergonovine, ergotamine, ergocornine, ergocristine, ergocryptine) of TEA was 62%. As proportions of TEA, ergonovine, ergotamine, ergocornine, ergocristine and ergocryptine constituted 7%, 26%, 5%, 18%, and 5%, respectively.

The analyzed TEA concentrations were 37%, 28%, 21% and 15% lower than the targeted contents in diets Ergot 1 to 4, respectively, whereas the corresponding key alkaloid concentrations were 36%, 26%, 20% and 14% smaller (Table 2).

**Table 1:** Analyzed composition of the ergoty rye batch No. 15 ^a^.

Crude Nutrients (g/kg) ^b^		Alkaloids (mg/kg) ^b^	
Crude ash	28.8	Ergonovine	31.9
Crude protein	183.7	Ergometrinine	7.5
Crude fat	321.8	Ergotamine	113.4
Crude fiber	203.8	Ergotaminine	38.0
Starch	32.9	Ergocornine	23.7
Sugar	14.2	Ergocorninine	10.2
Fatty acid composition (g/100 g crude fat)		Ergocristine	80.1
Caprylic acid (C8:0)	1.4	Ergocristinine	22.3
Lauric acid (C12:0)	2.8	Ergocryptine	22.3
Myristic acid (C14:0)	0.5	Ergocryptinine	19.9
Palmitic acid (C16:0)	30.2	Ergosine	54.8
Palmitoleic acid (C16:1)	3.3	Ergosinine	11.7
Stearic acid (C18:0)	7.8	Total alkaloids ^c^	435.8
Oleic acid (C18:1)	20.6	Key alkaloids ^d^	271.4
Linoleic acid (C18:2)	18.2		
Linolenic acid (C18:3)	0.4		
Arachidic acid (C20:0)	1.2		
Behenic acid (C22:0)	0.3		
Erucic acid (C22:1)	0.2		
Ricinoleic acid (12–OH–C18:1)	7.4		
Lignoceric acid (C24:0)	0.1		

^a^: Ergot batch contained 45.2% ergot and 54.8% rye; ^b^: Based on a dry matter content of 880 g/kg; ^c^: Sum of ergonovine, ergotamine, ergocornine, ergocristine, ergocryptine, ergosine and of their -inine isomers; ^d^: Sum of ergonovine, ergotamine, ergocornine, ergocristine, ergocryptine.

**Table 2:** Composition of the experimental diets (g/kg air dry feed).

	Diet				
	Control	Ergot 1	Ergot 2	Ergot 3	Ergot 4
Ingredients					
Wheat	417	417	417	417	417
Barley	146.2	146.2	146.2	146.2	146.2
Ergot batch 15 (see Table 1)	0	2.2	22.2	33.2	44.2
Rye	66.4	64.2	44.2	33.2	22.2
Soy meal	274.5	274.5	274.5	274.5	274.5
Soy oil	40	40	40	40	40
Di-Calcium phosphate	30.1	30.1	30.1	30.1	30.1
Calcium carbonate	7.8	7.8	7.8	7.8	7.8
Sodium chloride	4.8	4.8	4.8	4.8	4.8
l-lysine-HCl	1.1	1.1	1.1	1.1	1.1
l-threonine	0.8	0.8	0.8	0.8	0.8
dl-methionine	1.3	1.3	1.3	1.3	1.3
Premix ^a^	10	10	10	10	10
Calculated composition					
Crude protein	195	195	195	195	195
Crude fat	55.8	55.8	55.8	55.8	55.8
AME_N_ (MJ/kg)	11.88	11.9	11.9	11.9	11.9
Lysine	11	11	11	11	11
Methionine + cystine	7.5	7.5	7.5	7.5	7.5
Methionine	4.6	4.6	4.6	4.6	4.6
Threonine	7.5	7.5	7.5	7.5	7.5
Tryptophan	2.3	2.3	2.3	2.3	2.3
Calcium	11	11	11	11	11
Total phosphorus	9	9	9	9	9
Sodium	2	2	2	2	2
Total alkaloids ^b^ (mg/kg)	0.0	1.0	9.7	14.5	19.3
Key alkaloids ^c^ (mg/kg)	0.0	0.6	6.0	9.0	12.0
Analyzed composition					
Dry matter	888	890	880	885	889
Crude protein	190	192	189	193	191
Alkaloids (mg/kg)					
Ergonovine	<d.l.	0.05	0.45	0.81	1.05
Ergometrinine	<d.l.	0.01	0.10	0.18	0.24
Ergotamine	<d.l.	0.15	2.16	3.08	4.54
Ergotaminine	<d.l.	0.06	0.68	1.02	1.31
Ergocornine	<d.l.	0.03	0.35	0.67	0.83
Ergocorninine	<d.l.	0.01	0.19	0.22	0.25
Ergocryptine	<d.l.	0.04	0.39	0.64	0.94
Ergocryptinine	<d.l.	0.02	0.27	0.44	0.59
Ergocristine	<d.l.	0.12	1.08	1.96	3.01
Ergocristinine	<d.l.	0.04	0.28	0.61	0.82
Ergosine	<d.l.	0.08	0.80	1.36	2.23
Ergosinine	<d.l.	0.03	0.21	0.40	0.58
Total alkaloids ^b^	<d.l.	0.63	6.95	11.39	16.37
Key alkaloids ^c^	<d.l.	0.38	4.43	7.17	10.36

^a^: Provided per 1 kg diet: 1.5 g Ca; 1.5 g Na; 12,000 I.E. vitamin A; 3,000 I.E. vitamin D3; 40 mg vitamin E; 2 mg vitamin B1; 8.5 mg vitamin B2; 6 mg vitamin B6; 25 µg vitamin B12; 3 mg vitamin K3; 50 mg nicotinic acid; 15 mg Ca-panthotenate; 200 µg biotin; 500 mg choline chloride; 60 mg Fe; 12 mg Cu; 110 mg Mn; 80 mg Zn; 1.6 mg J; 0.32 mg Se; ^b^: Sum of ergonovine, ergotamine, ergocornine, ergocristine, ergocryptine, ergosine and of their -inin-isomeres; ^c^: Sum of ergonovine, ergotamine, ergocornine, ergocristine, ergocryptine; d.l.: detection limit.

### 2.2. Feed Intake, Growth Performance and Alkaloid Exposure

**Figure 1:**
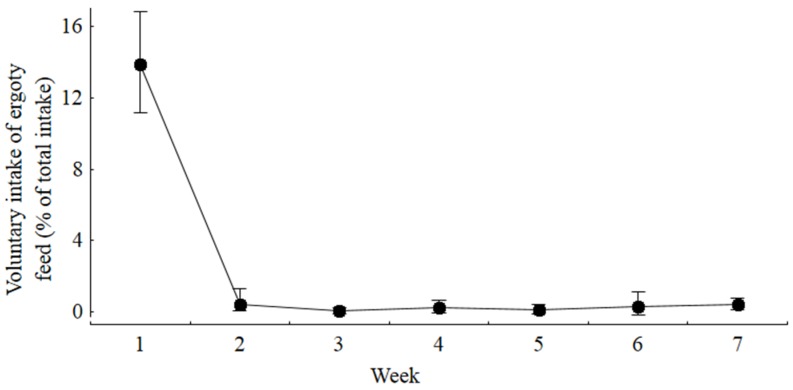
Proportion of ergot contaminated diet voluntarily consumed by ducks offered diets containing either 0 or 20 g ergot/kg diet at the same time for free choice (expressed as percentage of total feed intake). Proportion in week 1 differed significantly from the corresponding proportions in all other experimental weeks (*n* = 9, *p* < 0.05). Symbols denote mean values and whiskers denote minimum and maximum values.

Voluntary feed intake decreased significantly and continuously during the first week of the experiment as the ergot content of the diets increased from 0.1, 1.0, and 1.5 to 2.0 and accounted for 91%, 72%, 59% and 53% of feed consumed compared to the control group (Table 3). The choice feeding group consumed the same total amount of feed as the control group. Ducks of this group selected approximately 86% of the control and 14% of the Ergot 4 diet. Ducks of this group effectively learned to differentiate between both diets from the second week of the experiment onwards when the voluntary intake of the Ergot 4 diet declined to less than 2% and remained at this level until the end of the study (Figure 1).

The feed intake depressing effects of dietary ergot contamination was even more pronounced in week 2 of the experiment when ducks of Groups Ergot 3 and 4 consumed 51% and 61% less feed than the control group, respectively (Table 3).

In spite of the ergot related feed intake decrease the exposure of the ducks to total ergot alkaloids increased linearly with the ergot content of the diets (Table 4).

**Table 3:** Performance of ducks exposed to diets containing increasing ergot levels (*n* = 9).

Group	Ergot (g/kg Diet)	Feed Intake (g/d)		Live Weight Gain (g/d)		Feed to Gain Ratio (g/g)	
		Day 1–7	Day 8–14	Day 1–7	Day 8–14	Day 1–7	Day 8–14
Control	0	35.1 ^e^	98.5 ^d^	25.7 ^e^	66.6 ^e^	1.370 ^a^	1.479
Ergot 1	1	31.8 ^d^	94.7 ^d^	23.4 ^d^	62.5 ^d^	1.368 ^a^	1.515
Ergot 2	10	25.4 ^c^	78.7 ^c^	17.2 ^c^	54.4 ^c^	1.475 ^b^	1.448
Ergot 3	15	20.8 ^b^	48.7 ^b^	12.5 ^b^	33.3 ^b^	1.672 ^c^	1.466
Ergot 4	20	18.7 ^a^	38.1 ^a^	10.7 ^a^	24.8 ^a^	1.766 ^c^	1.534
Choice ^g^	0 and 20	37.3 ^f^	98.6 ^d^	24.5 ^d^	65.1 ^d^	1.524 ^b^	1.516
*p*-value		<0.001	<0.001	<0.001	<0.001	<0.001	0.065
PSEM		0.5	1.6	0.5	1.0	0.035	0.022

^a–f^: Values with no common superscripts are significantly different within columns (*p*<0.05); ^g^: Choice feeding group: Ducks were offered the diets containing 0 and 20 g ergot/kg diet at the same time for free choice; PSEM = pooled standard error of means.

**Table 4:** Mean daily exposure of ducks to total alkaloids and key alkaloids (µg/kg body weight) (*n* = 9).

Group	Ergot (g/kg Diet)	Total Alkaloids		Key Alkaloids	
		Day 1–7	Day 8–14	Day 1–7	Day 8–14
Control	0	0	0	0	0
Ergot 1	1	147 ^a^	137 ^b^	89 ^a^	83 ^b^
Ergot 2	10	1533 ^c^	1497 ^c^	976 ^c^	953 ^c^
Ergot 3	15	2408 ^d^	2143 ^d^	1515 ^d^	1348 ^d^
Ergot 4	20	3331 ^e^	2877 ^e^	2108 ^e^	1821 ^e^
Choice ^f^	0 and 20	530 ^b^	14 ^a^	335 ^b^	9 ^a^
*p*-value		<0.001	<0.001	<0.001	<0.001
PSEM		45	32	28	20

Statistics was performed without the control group; PSEM = pooled standard error of means; ^a–e^: Values with no common superscripts are significantly different within columns (*p* < 0.05); ^f^: Choice feeding group: Ducks were offered the diets containing 0 and 20 g ergot/kg diet at the same time for free choice.

Live weight gain reflected the significance relationships as described for feed intake, and this decreased significantly with increasing dietary ergot contamination. Although the lowest ergot contamination level resulted in a significant adverse effect for feed intake and live weight gain, the corresponding feed to gain ratio started to increase significantly at a higher ergot inclusion (Ergot 2) during week 1, whereas no significant effect was observed during week 2.

**Figure 2:**
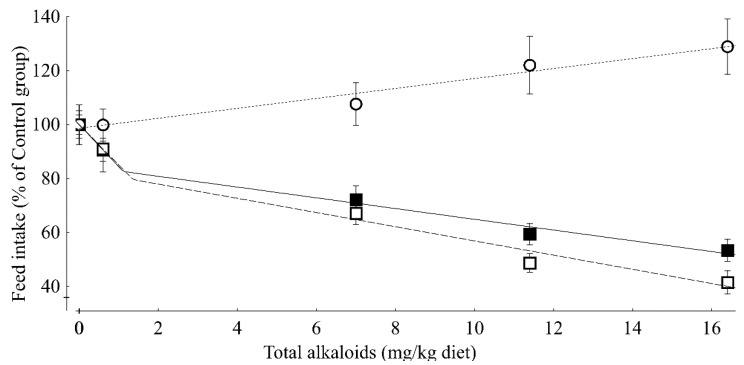
Effects of increasing concentrations of total ergot alkaloids in duck diets on feed intake(—■—, y = ((100−15.7x) (x ≤ 1.1)) + ((84.8−1.99x) (x > 1.1)), *r*² = 0.993, RSD = 2.3%), live weight gain (---□---, y = ((100−15.0x) (x ≤ 1.4)) + ((83.2−2.63x) (x > 1.4)), *r*² = 0.989, RSD = 3.6%) and feed to gain ratio (∙∙∙Ο∙∙∙, y = 100 + 1.8x, *r*² = 0.966, RSD = 2.4%)) during week 1 of the experiment (expressed as percentage of the non-exposed control group). The lowest dietary concentrations of total ergot alkaloids (TEA) causing a significant decrease in feed intake and live weight gain (LOAL) amounted to 0.6 mg/kg, whereas feed to gain ratio responded with a significant increase at 7 mg TA/kg diet (*n* = 9). Symbols denote mean values and whiskers denote the standard deviation. For significance relationships see Table 3.

Performance data from week one of the experiment were further evaluated regressively (Table 3). Generally, feed intake and live weight gain decreased with increasing dietary TEA, while feed to gain ratio increased. The feed intake and live weight gain decreases seemed to be more pronounced at the lowest TEA concentration of 0.6 mg/kg than at all higher concentrations, and suggested linear regressions with distinct structure breaks (Figure 2). Interpreting the results of the broken-line regressions revealed a decrease of 15.7% and 15% per each 1 mg increase of TEA per kg diet up to the break-points of 1.1 and 1.4 mg TEA/kg diet, respectively, whereas the corresponding decreases beyond these break-points were 1.99% and 2.63% per one mg TEA/kg. Such a distinct structure break could not be identified for feed to gain ratio, which continuously increased at 1.8% per one mg TEA increase/kg diet (Figure 2).

Although no duck died during the first two weeks of the experiment, the magnitude of the decrease in feed intake by 50% and more recorded in groups Ergot 3 and 4 prompted us to terminate the experiment for these two groups after two weeks. For the remaining four groups, we terminated the experiment after the scheduled period of seven weeks.

Although the decrease in feed intake started to be significant at the lowest ergot inclusion level of 1 g/kg during the first two weeks of the experiment in later periods (Day 15–49), the ergot-related decrease occurred at the 10-fold higher ergot content of 10 g/kg diet (Table 5). Moreover, as live weight gain was not affected by the ergot level the feed to gain ratio was even significantly improved by 8% in this period. The choice feeding group performed at the same level as the control group. The ergot exposure increased proportionally with the ergot content of the diets in the later period of the experiment. Alkaloid exposure increased from week 1 to week 2 and decreased thereafter within the same dietary ergot level (Table 4 and Table 6).

**Table 5:** Performance of ducks exposed to diets containing increasing ergot levels (*n* = 9).

Group	Ergot (g/kg diet)	Feed Intake (g/d)			Live Weight Gain (g/d)			Feed to Gain Ratio (g/g)		
		Day 1–14	Day 15–49	Day 1–49	Day 1–14	Day 15–49	Day 1–49	Day 1–14	Day 15–49	Day 1–49
CON	0	66.8 ^c^	215.4 ^b^	172.9 ^b^	46.2 ^d^	78.0	68.9 ^b^	1.448 ^a^	2.761 ^b^	2.509 ^b^
Ergot 1	1	63.2 ^b^	208.9 ^b^	167.3 ^b^	42.9 ^b,c^	77.2	67.4 ^a,b^	1.474 ^a^	2.707 ^b^	2.482 ^b^
Ergot 2	10	52.1 ^a^	194.2 ^a^	153.6 ^a^	35.8 ^a^	76.6	64.9 ^a^	1.454 ^a^	2.538 ^a^	2.367 ^a^
Choice ^e^	0 and 20 ^e^	68.0 ^c^	210.4 ^b^	169.7 ^b^	44.8 ^c,d^	76.9	67.7 ^b^	1.518 ^b^	2.737 ^b^	2.507 ^b^
*p*-value		<0.001	<0.001	<0.001	<0.001	0.828	0.029	0.001	<0.001	<0.001
PSEM		1.0	2.9	2.3	0.7	1.1	0.9	0.012	0.028	0.021

^a–d^: Values with no common superscripts are significantly different within columns (*p* < 0.05); ^e^: Choice feeding group: Ducks were offered the diets containing 0 and 20 g ergot/kg diet at the same time for free choice; PSEM = pooled standard error of means.

**Table 6:** Daily exposure of ducks to total alkaloids and key alkaloids in dependence of increasing dietary ergot contents (*n* = 9).

Group	Ergot (g/kg Diet)	Key Alkaloids (µg/kg Body Weight)			Total Alkaloids (µg/kg Body Weight)		
		Day 1–14	Day 15–49	Day 1–49	Day 1–14	Day 15–49	Day 1–49
Control	0	0	0	0	0	0	0
Ergot 1	1	68 ^a^	40 ^b^	38 ^b^	112 ^a^	66 ^b^	62 ^b^
Ergot 2	10	754 ^c^	453 ^c^	413 ^c^	1185 ^c^	712 ^c^	649 ^c^
Choice ^d^	0 and 20 ^d^	137 ^b^	3 ^a^	23 ^a^	216 ^b^	4 ^a^	36 ^a^
*p*-value		<0.001	<0.001	<0.001	<0.001	<0.001	<0.001
PSEM		5	2	2	8	3	4

^a–c^: Values with no common superscripts are significantly different within columns (*p* < 0.05); ^d^: Choice feeding group: Ducks were offered the diets containing 0 and 20 g ergot/kg diet at the same time for free choice; PSEM = pooled standard error of means; Statistics was performed without control group.

### 2.3. Blood Clinical-Chemistry and Hematology

Gamma-glutamyl-transferase (GGT) was significantly increased approximately twofold in ducks of group Ergot 2 when compared to groups Control and Ergot 1 and the choice feeding group (Table 7). The activity measured in the latter group was also significantly higher than in Group Ergot 1. In contrast, the activities of glutamate dehydrogenase (GLDH) and aspartate aminotransferase (ASAT) remained uninfluenced by treatment. Although glucose concentration was not affected by treatments, the albumin concentration was significantly higher in the choice feeding group and in Group Ergot 1 compared to the control group.

Monocyte proportions were significantly lower in all treatment groups compared to the control group, whereas all other leukocytes remained uninfluenced (Table 8).

### 2.4. Organ Weights

Liver weight was significantly increased by 10% in ducks of group Ergot 2 compared to the control group, whereas a decrease by 12% was recorded for the livers of the choice feeding group (Table 9). Similarly, the weight of the emptied small intestine was significantly lower in the choice feeding group compared to all other groups, which insignificantly differed from each other. The weight of the abdominal fat decreased as the ergot content increased by 10% and 17% in groups Ergot 1 and 2 compared to the control group, respectively. The choice feeding group reached the same level of abdominal fat weight as the control group. The gizzard weight was significantly enhanced by approximately 10% in the Ergot 2 group compared to the control group, whereas no significant differences were detected for the other groups. Although similar relative mean value differences were observed for the glandular stomach, they were not significant. The relative weights of the heart, spleen and bursa cloacalis were not influenced by treatments.

### 2.5. Alkaloid Residues in Physiological Specimens

Alkaloid concentrations in liver, breast meat and serum were lower than 5 ng/g. Bile collected from ducks of group Ergot 2 was the only matrix positive for ergot alkaloids with ergonovine being the only detected alkaloid. The mean concentration was 40 ng/g and varied from 35 to 44 ng/g and corresponded to a mean ratio of 0.08 (0.05–0.1) between bile and feed concentration of ergonovine.

## 3. Discussion

The most striking observation of the present experiment was the pronounced sensitivity of ducks to ergot alkaloids. Ducks responded with adverse effects even to the lowest TEA concentration of 0.6 mg per kg diet. These results suggested that ducks obviously respond more sensitive to TEA than other poultry species.

**Table 7:** Blood clinical-chemistry of ducks fed diets with increasing ergot levels (*n* = 18, day 49).

Group	Ergot (g/kg Diet)	Gamma-Glutamyl-Transferase (U/L)	Glutamate Dehydrogenase (U/L)	Aspartate Aminotransferase (U/L)	Glucose (mMol/L)	Albumin (g/dL)
Control	0	5.1 ^a,b^	6.1	49.1	10.1	1.4 ^a^
Ergot 1	1	3.9 ^a^	7.2	46.3	11.1	1.6 ^b,c^
Ergot 2	10	9.8 ^c^	7.0	51.8	10.0	1.5 ^a,b^
Choice ^d^	0 and 20 ^d^	5.8 ^b^	5.5	50.9	9.9	1.7 ^c^
*p*-value		<0.001	0.330	0.643	0.161	0.002
PSEM		0.7	0.8	3.3	0.4	0.1

^a–c^: Values with no common superscripts are significantly different within columns (*p* < 0.05); ^d^: Choice feeding group: Ducks were offered diets containing 0 and 20 g ergot/kg diet at the same time for free choice; PSEM = pooled standard error of means.

**Table 8:** White differential blood count (%) of ducks fed diets with increasing ergot levels (*n* = 18, day 49).

Group	Ergot (g/kg Diet)	Lymphocytes	Monocytes	Heterophiles	Eosinophiles	Basophils	Heterophiles/Lymphocytes
Control	0	47.7	6.7 ^b^	42.2	1.1	2.3	0.9
Ergot 1	1	52.4	2.8 ^a^	40.2	0.8	3.9	0.8
Ergot 2	10	49.9	1.4 ^a^	44.8	0.4	3.6	0.9
Choice ^c^	0 and 20 ^c^	47.0	2.3 ^a^	47.4	0.6	2.7	1.0
*p*-value		0.420	<0.001	0.198	0.197	0.139	0.225
PSEM		2.5	0.6	2.5	0.2	0.5	0.1

^a,b^: Values with no common superscripts are significantly different within columns (*p* < 0.05); ^c^: Choice feeding group: Ducks were offered diets containing 0 and 20 g ergot/kg diet at the same time for free choice; PSEM = pooled standard error of means.

**Table 9:** Relative weight of organs and abdominal fat (g/kg body weight) of ducks fed diets with increasing ergot levels (*n* = 18, day 49).

Group	Ergot (g/kg Diet)	Liver	Heart	Small Intestine	Spleen	Abdominal Fat	Gizzard	Glandular Stomach	*Bursa Cloacalis*
Control	0	20.5 ^b^	5.5	20.7 ^b^	0.6	7.1 ^a,b^	29.3 ^a^	2.8	1.1
Ergot 1	1	21.5 ^b,c^	5.2	19.2 ^b^	0.6	6.4 ^a^	29.1 ^a^	2.8	0.9
Ergot 2	10	22.5 ^c^	5.5	19.5 ^a,b^	0.6	5.9 ^a^	31.8 ^b^	3.1	1.0
Choice ^d^	0 and 20 ^d^	18.1 ^a^	5.4	18.5 ^a^	0.6	7.8 ^b^	28.7 ^a^	2.8	1.0
*p*-value		<0.001	0.764	0.028	0.655	0.039	0.022	0.178	0.169
PSEM		0.6	0.2	0.5	0.0	0.5	0.8	0.1	0.1

^a–c^: Values with no common superscripts are significantly different within columns (*p* < 0.05); ^d^: Choice feeding group: Ducks were offered the diets containing 0 and 20 g ergot/kg diet at the same time for free choice; PSEM = pooled standard error of means.

### 3.1. Dietary Ergot Alkaloids

The analyzed TEA in the diets were lower than those calculated based on the analysis of the ergoty rye mixed into the diets which might be due to several reasons. The generally lower TEA concentrations of the diets might be caused by matrix effects of the diet which could give rise to destruction of alkaloids by interactions with diet constituents. Other reasons might include a dust associated disappearance of TEA during diet mixing or handling. Time effects might also be possible due to the time lag between designing and performing the experiment. The deviation from the targeted TEA concentrations decreased with increasing dietary ergot inclusion rates suggesting that sampling error might also play a role that is known to increase with decreasing concentrations of micro-components of a diet.

### 3.2. Duck Experiment

Our experiment demonstrated that ducks responded very sensitively to the presence of ergot alkaloids in feed with a significant decrease in voluntary feed intake even at the lowest TEA concentration of 0.6 mg/kg diet. Due to this pronounced feed intake-depressing effect, live weight gain was influenced in a similar manner. Although both feed intake and live weight gain responded in a similar direction, the resulting feed to gain ratio increased at the same time during week 1 of the experiment. Therefore, the nutrient and energy utilization was dose dependently compromised during the initial period of the experiment. In contrast, during week 2 of the experiment, the feed to gain ratio remained uninfluenced and was even decreased during the later periods of the experiment for the groups remaining in the experiment.

The ergot-associated decrease in feed intake might have been caused by several factors. Generally, common features of plant alkaloids are their bitter taste and the pharmacological activity. Plant alkaloids serve as a chemical defense against herbivory [6] and it is assumed that the ecological role of ergot alkaloids is also to protect the fungi from consumption by vertebrate and invertebrate animals [7]. Ducks and chickens, compared to pigeons, seem to be particularly sensitive to a bitter taste as indicated by a pronounced rejection of a solution of quinine hydrochloride, a model substance for bitterness, at low concentrations [8]. Male geese (another waterfowl closely related to ducks), when compared to turkeys, Japanese quail and chickens, had a more pronounced response with a decrease in voluntary intake of quinine-containing diets in a dose-dependent manner [9]. In discussing the feed intake depressing effects of ergot, the presence of further substances with potential anorectic or other toxic acting substances in ergot, such as ricinoleic acid, need to be considered. As ricinoleic acid increased with dietary ergot and consequently with TEA, it is impossible to assign individual or interactive effects conclusively. However, sub-acute toxicity studies with mice and rats fed graded levels of castor oil processed from the castor bean plant, *Ricinus communis* L, which contains approximately 90% ricinoleic acid, revealed that dietary proportions of 10% (100g/kg) castor oil did not cause adverse effects [10]. That ricinoleic acid might contribute only partly to the overall toxicity of ergot might be deduced from stepwise regressions of TEA and ricinoleic acid from ergot on the live weight gain of piglets. Although TEA alone could explain 82% of the variation in live weight gain, the additional inclusion of ricinoleic acid increased the variation explained to only 86% [11]. Because of the low toxicity in rodents and the rather small contribution of ricinoleic acid to the overall toxicity of ergot, a rather low effect might be deduced for the present experiment where the highest dietary ricinoleic acid concentration amounted to 1.1 g/kg.

The chicken is generally believed to avoid toxin-containing diets that had previously caused illness, disturbances or discomfort [12]. This general effect might be caused by metabolic signals due to ingesting the substance under question besides, or in addition to taste aversion. In the present experiment, we found a pronounced decrease in feed intake of *ad libitum* fed ducks when no chance for avoiding the contaminated diets was offered, while choice fed ducks effectively learned to avoid the contaminated diet within one week in spite of weekly changes to the positions of the troughs containing the uncontaminated and the ergot-containing diet. Besides the bitter taste acting at the ingestive phase, metabolic alterations caused by the ergot alkaloid interactions with serotoninergic, dopaminergic and adrenergic receptors might also contribute to the post-ingestive modulation of voluntary feed intake. Ergot alkaloids are largely capable of permeating the blood-brain barrier [13], which supports the view that decrease in feed intake in *ad libitum* fed ducks and ergot-containing feed rejection in choice fed ducks might be mediated by central-nervous signals. Although we failed to detect ergot alkaloids in systemic blood, we confirmed the presence of ergonovine in bile, which indicates at least a portal absorption while a small fraction might have entered the systemic circulation and eventually the blood-brain barrier.

Recently, it was shown that colon and liver cell lines (HT-29, HepG2) are capable of intense hydroxylation at the peptide moiety while the toxico-dynamically relevant ergoline structure remained untouched [14]. Thus, the peptide ergot alkaloids ergotamine/ergotaminine and ergocristine/ergocristinine were metabolized in this way while the lysergic acid amide derivatives ergonovine/ergonovinine were neither taken up by the cells nor metabolized [14,15]. The unaltered structure of ergonovine might explain, at least in part, why we could detect solely this ergot alkaloid in bile, as our HPLC-method barely detects the free forms of ergot alkaloids, while metabolized forms including lysergic acid escape detection. However, the fact that ergonovine/ergonovinine were not taken up either by intestinal or hepatic cell lines in these *in vitro* experiments raises the question on the reasons for the presence of ergonovine in bile in our *in vivo* study. It might be hypothesized that only ergonovine could be detected amongst the ergot alkaloids and solely in bile due to absorption of small amounts over a longer period of time in combination with its accumulation and its poor metabolism, leaving it detectable by our HPLC-method.

The liver not only plays a role in metabolizing and excretion of ergot alkaloids as discussed above, but has also been suggested as a primary target of portally delivered toxins.

Activities of enzymes in blood that are more or less indicative for hepatic lesions such as γ-glutamyl transferase (GGT), glutamate dehydrogenase (GLDH) and the less specific aspartate aminotransferase (ASAT) have been shown to respond either not at all (GLDH, GGT) or with an inconsistent and not always reproducible increase (ASAT) in piglets fed diets varying largely in the TEA between 3 and 21 mg/kg [16,17,18,19,20], whereas in calves, fattening bulls and dairy cows no effects on these enzyme activities could be detected [21,22,23]. Also to be considered in evaluating these effects are the differences in the pattern of individual alkaloids which are summed up by the TEA content, which was demonstrated to play a role for feed intake and live weight gain but was less important for liver lesions [17,19]. Broilers were shown to respond with an increase in GGT and ASAT activity when fed diets with TEA of 3 and 11 mg/kg diet [20]. In our study, GGT activity in ducks increased significantly at a dietary TEA of 7 mg/kg diet, which is approximately 10-fold higher than the diet concentration found to be effective for decreasing feed intake, indicating that the liver responds less sensitively than feed intake in ducks, also. Interestingly, and in contrast to reports of ergot-associated decreases in blood albumin concentration in piglets and broiler chickens [19,20], ducks responded with an increase even at the lowest diet concentration of 0.6 mg TEA/kg. As albumin concentration in blood not only reflects the liver function with regard to protein synthesis function but might also be affected by hemo-concentration or -dilution, the contradictory results should not be overemphasized.

That the liver obviously responds to TEA at higher dietary concentrations than feed intake or live weight gain is further substantiated by the results of liver function tests. These tests are based on the hepatic cytochrome P450 (CYP1A2)-catalyzed conversion of stable isotope-labeled substrates to labeled CO_2_, which can be measured in breath. These tests indicated LOAELs of 17 and 21 mg TEA/kg diet for male and female piglets, respectively [18] while a literature compilation on the relationships between TEA content of the diet and performance revealed an incremental decrease of 0.9% and 1.24% per each 1 mg increase of TEA per kg diet when feed intake and live weight gain were used as response criteria [16]. Based on the corresponding decreases of 15.7% and 15.0% for TEA concentrations ≤1.1 and 1.4 mg TEA/kg, and at 1.99% and 2.63% for TEA concentrations higher than these break-points, the ducks seem to respond more sensitively to dietary ergot alkaloids than piglets.

Whether the ergot-associated increase in liver weight relative to body weight is a direct consequence of the toxic action of ergot alkaloids or due to a retardation of muscle growth relative to the dynamics of weight development of inner organs as a reflection of the ontogenetic allometry cannot be answered conclusively as further parameters such as histopathological lesions were not recorded. Similarly, the ergot induced increase of the relative gizzard weight might be explained in this way although other digestive organs appeared to be unaffected.

The only conspicuous difference in white blood count was the ergot-related decrease in the proportions of monocytes, which might hint at an effect of ergot alkaloids in modulation of immune responses. Subcutaneous ergotamine tartrate doses equal or higher than 2 mg/kg body weight resulted in the secretion of pro-inflammatory cytokines by LPS stimulated murine splenocytes and macrophages, while differential white blood count remained unaffected [24]. In steers, the LPS-induced acute phase response was attenuated by intravenous administration of ergotamine, which was supposed to result from the ergotamine-associated cortisol increase [25]. Thus, the relevance and the nature of the ergot-associated decrease in the monocyte proportions observed in our experiment requires further experimental consideration. The general conclusion that poultry tolerates higher levels of ergot alkaloids than other non-ruminant livestock [2] was drawn by deriving a NOAEL of 1.4 mg TEA/kg diet based on reviewing the literature published since the last EFSA-opinion on ergot alkaloids in 2005. However, mainly chickens were considered in the latest EFSA-opinion while no experiments on ducks were available at that time. As the lowest dose tested in our experiments caused adverse effects, we could not derive a NOAEL. Thus, the NOAEL for ducks is lower than 0.6 mg TEA/kg and consequently also markedly lower than that of 1.4 mg TEA/kg diet for chickens, as suggested by EFSA [2].

**Figure 3:**
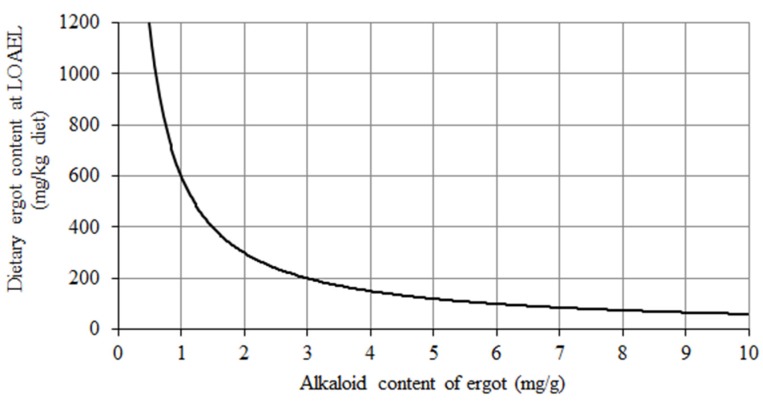
Estimation of the ergot proportion in diets for ducks (mg/kg diet) where the LOAEL of 0.6 mg TEA/kg diet is reached in dependence on varying TEA contents of ergot (mg/g ergot).

In view of the unreliability of the current feed safety regulations regarding the upper limit of 1000 mg ergot (*C. purpurea*) per kg unground cereal grains (=0.1%), as specified by Directive 2002/32/EC, and the significant variation in TEA content of ergot as reviewed by EFSA [2], a risk evaluation for the duck covering this variation and considering the LOAEL of 0.6 mg TEA/kg diet was performed to identify those dietary ergot levels where this LOAEL is reached for a particular TEA content of ergot (Figure 3). As we failed to derive a NOAEL, the estimated dietary ergot contents indicate ergot levels where adverse effects already can be expected. For example, if the ergot alkaloid content of ergot reaches approximately 0.6 mg/g the permitted ergot content of 1000 mg/kg feed would also be exceeded. However, this is not a realistic scenario as the upper limit applies for pure (unground) cereal grains, which do not constitute the whole diet of a duck (see also Table 2). Rather, the cereal content of a duck diet varies from a few percent up to more than 50%, which also needs to be considered when evaluating the risk of ergot contaminated cereal grains for this poultry species (Figure 3). For example, the LOAEL of 0.6 mg TEA/kg diet would correspond to a dietary ergot content of 600 mg/kg diet when the diet would consist of 100% of cereal grains contaminated by ergot with a TEA content of 1 mg/g ergot (Figure 3). If the same grain batch would be incorporated into the diet at a proportion of 50%, the TEA content of the ergot could approximate 2 mg/g. More generally expressed, the area to the right of the curve in Figure 3 represents all combinations of TEA content of ergot and dietary ergot content, which would exceed the LOAEL. Again, as the curve was constructed based on LOAEL instead of the NOAEL, the combinations of dietary ergot content and TEA content of ergot, which would result in safe dietary TEA levels, are not known so far but are within the area left of the curve.

## 4. Experimental Section

### 4.1. Experimental Design and Diets

The experimental design was a dose-response study. An ergoty rye batch containing 45.2% ergot and 54.8% rye was used as ergot source. The control diet was prepared without ergot, and the diets Ergot 1 to 4 contained 1, 10, 15 and 20 g ergot per kg diet, respectively, and corresponded to 2.2, 22.2, 33.2 and 44.2 g of the ergoty rye batch (Table 1 and Table 2). All experimental diets were formulated to be isoenergetic and isonitrogen based on the main components wheat, barley and soybean meal.

An additional choice feeding group was offered the control diet along with the diet with the highest ergot content (20 g/kg) (Table 10) at the same time.

**Table 10:** Experimental design.

Group	Ergot (g/kg Diet)	Calculated Alkaloid Concentrations (mg/kg Diet)	
		Total	Key ^a^
Control	0	0.0	0.0
Ergot 1	1	1.0	0.6
Ergot 2	10	9.7	6.0
Ergot 3	15	14.5	9.0
Ergot 4	20	19.3	12.0
Choice ^b^	0 and 20	0.0 and 19.3	0.0 and 12.0

^a^: Sum of ergonovine, ergotamine, ergocornine, ergocristine and ergocryptine; ^b^: Choice feeding group: Ducks were offered the diets containing 0 and 20 g ergot/kg diet at the same time for free choice.

### 4.2. Growth Experiment

The study covered the period from hatch until day 49 of age. One day-old unsexed Pekin ducks were obtained from the breeding company Stolle GmbH, Westerschep, Germany and randomly assigned to the six treatment groups (Table 10). Six ducklings were placed in each of the 54 wood shave bedded floor pens with the dimensions of 1 m × 1.2 m, except that the choice feeding group occupied two of these pens per simultaneous replication. In total, each treatment group was replicated nine times. Therefore, each treatment comprised a total of 54 ducks. Feed and water were offered for *ad libitum* consumption. Temperature and lighting regimes were in accordance with the recommendations of the breeder.

Duck weight and consumed feed were determined weekly. At the end of the study, after the final weighing, 2 ducks per pen were slaughtered by cutting the neck vessels after manual stunning (*n* = 18 per group). Mixed trunk blood was collected for preparing blood smears and serum. Liver, heart, small intestine, spleen, abdominal fat, gizzard, glandular stomach and *Bursa cloacalis* were dissected and weighed. In addition, breast meat, liver, serum and bile were frozen for later analysis for alkaloid residues.

Treatments and experiments were conducted according to the European Community regulations concerning the protection of experimental animals and were approved by the State Bureau for Consumer Protection and Food Safety of Lower Saxony (LAVES) in Oldenburg, Germany.

### 4.3. Analyses

The ergot batch used was analyzed for dry matter, crude protein, crude ash, crude fat, starch and sugar according to the official standard methods of the Association of German Agricultural Research and Investigation Institutions (VDLUFA) [26]. The experimental diets were analyzed for dry matter and crude protein. The ergot batch was further analyzed for fatty acids using gas chromatography, as published in detail earlier [27,28].

Ergot alkaloids (ergonovine, ergocornine, ergotamine, α-ergocryptine, ergosine, ergocristine and their -inine isomers) in ergot, diets, serum, bile, freeze-dried liver and breast meat were analyzed by adapting an HPLC based method [29], as described in detail elsewhere [23]. The limit of quantification (LOQ) was 5 ng/g at a sample size of 5 g for all specimens. The mean recovery rates varied between 45% and 139% depending on matrix and specific alkaloid [23]. Measured alkaloid concentrations were not corrected for recovery. Ergonovine, ergotamine, ergocristine, ergocornine and ergocryptine standards were commercially available for their identification (Sigma-Aldrich Chemie GmbH, Buchs, Switzerland). These standards were also used for the identification of their corresponding -inine isomers while ergosine and its isomer were identified through their retention time [30]. Finally, the sum of all identified alkaloids (*-*ine and *-*inine isomers) is designated herein as total ergot alkaloids (TEA).

Serum clinical-chemical parameters were determined using test-kits supplied by Merck, Darmstadt, Germany: glutamate dehydrogenase (GLDH, EC 1.4.1.3, Merck-1-Test^®^, 1.03373), γ-glutamyltransferase (GGT, EC 2.3.2.2, Granutest^®^ 3, 1.12189.0001), aspartate aminotransferase (ASAT, EC 2.6.1.1, Granutest^®^ 3, 12150), glucose (Granutest^®^ 100, 1.12193) and albumin (Granutest^®^, 1.14819.0001).

Blood smears were prepared and stained using Wright-Giemsa stain (Sigma-Aldrich Chemie GmbH, Steinheim, Germany) for differentiating the white blood cells. Two hundred cells were counted using a light microscope (Carl Zeiss Microscopy GmbH, Jena, Germany) at a magnification of 100× and differentiated for heterophils, lymphocytes, monocytes, eosinophils, and basophils. Moreover, the ratio between heterophils and lymphocytes (H/L ratio) was calculated.

### 4.4. Calculations and Statistics

Daily feed intake was calculated by dividing the feed consumption per pen (difference between offered and back-weighed feed) by the number of days and ducks present in the respective period. Daily live weight gain was calculated as the difference between two weight records of each individual duck divided by the corresponding days. As feed intake could only be recorded on a pen basis, the live weight gain and the feed to live weight gain ratio were expressed on that basis as well; *i.e.*, the number of replications per treatment equaled nine for each of these three parameters. Mean daily exposure to alkaloids was obtained by multiplying the daily feed intake with the analyzed alkaloid concentration in feed and dividing by the corresponding mean live (body) weight.

Organ weights were related to body weight and expressed as g organ weight per kg body weight.

Data were analyzed according to a one-way factorial design of analysis of variance (ANOVA) according to the following model: y_ij_ = µ + a_i_ + e_ij_; where y_ij_ = jth observation subjected to treatment i; µ = overall mean; a_i_ = effect of treatment; e_ij_ = residual error.

In case of a significant treatment effect the individual mean value differences were examined for significance by using the Tukey test. All statistics were performed using the Statistica for the Windows Operating System, Version 7.1, 1995 (StatSoft Inc., Tulsa, OK, USA).

## 5. Conclusions

The exclusive detection of ergonovine amongst the ergot alkaloids in bile requires further experiments to clarify the fate of ergot alkaloids with special consideration of *in vivo* metabolism and analytical detectability of evolving metabolites.

The lowest TEA concentration of 0.6 mg per kg diet induced adverse effects in ducks suggesting that this poultry species is not protected by the current EU legislation (1 g ergot/kg unground cereal grains) when the natural variation in TEA content of ergot is taken into account.
